# A new 4-gene-based prognostic model accurately predicts breast cancer prognosis and immunotherapy response by integrating WGCNA and bioinformatics analysis

**DOI:** 10.3389/fimmu.2024.1331841

**Published:** 2024-02-02

**Authors:** Wenlong Chen, Yakun Kang, Wenyi Sheng, Qiyan Huang, Jiale Cheng, Shengbin Pei, You Meng

**Affiliations:** ^1^ Department of Thyroid and Breast Surgery, The Affiliated Suzhou Hospital of Nanjing Medical University, Suzhou Municipal Hospital, Gusu School, Nanjing Medical University, Suzhou, China; ^2^ Department of Breast Surgery, The First Affiliated Hospital of Nanjing Medical University, Nanjing, China; ^3^ Department of Breast Surgical Oncology, National Cancer Center/National Clinical Research Center for Cancer/Cancer Hospital, Chinese Academy of Medical Sciences and Peking Union Medical College, Beijing, China

**Keywords:** breast cancer, POLQ, prognostic signature, immune landscape, drug screening

## Abstract

**Background:**

Breast cancer (BRCA) is a common malignancy in women, and its resistance to immunotherapy is a major challenge. Abnormal expression of genes is important in the occurrence and development of BRCA and may also affect the prognosis of patients. Although many BRCA prognosis model scores have been developed, they are only applicable to a limited number of disease subtypes. Our goal is to develop a new prognostic score that is more accurate and applicable to a wider range of BRCA patients.

**Methods:**

BRCA patient data from The Cancer Genome Atlas database was used to identify breast cancer-related genes (BRGs). Differential expression analysis of BRGs was performed using the ‘limma’ package in R. Prognostic BRGs were identified using co-expression and univariate Cox analysis. A predictive model of four BRGs was established using Cox regression and the LASSO algorithm. Model performance was evaluated using K-M survival and receiver operating characteristic curve analysis. The predictive ability of the signature in immune microenvironment and immunotherapy was investigated. *In vitro* experiments validated POLQ function.

**Results:**

Our study identified a four-BRG prognostic signature that outperformed conventional clinicopathological characteristics in predicting survival outcomes in BRCA patients. The signature effectively stratified BRCA patients into high- and low-risk groups and showed potential in predicting the response to immunotherapy. Notably, significant differences were observed in immune cell abundance between the two groups. *In vitro* experiments demonstrated that POLQ knockdown significantly reduced the viability, proliferation, and invasion capacity of MDA-MB-231 or HCC1806 cells.

**Conclusion:**

Our 4-BRG signature has the potential as an independent biomarker for predicting prognosis and treatment response in BRCA patients, complementing existing clinicopathological characteristics.

## 1 Introduction

Breast cancer (BRCA) has overtaken lung cancer to become the world’s leading cancer and the most deadly malignancy among women (1). With the continuous progress of BRCA diagnosis and treatment technology, the 5-year survival rate of early BRCA patients can reach 95%, so early screening, diagnosis, and treatment of BRCA are the keys to a good prognosis (2). BRCA is a highly heterogeneous disease, and patients differ in their response to treatment and prognosis even if the clinical stage and pathological grade are the same. Although these molecular subtypes are widely used, the prognosis of BRCA cases of each subtype is still very different. Therefore, it is of great clinical significance to explore new prognostic features.

In cancer research, prognostic models are widely used to predict the prognosis of patients. Van De Vijver et al. first performed 70 genetic signatures that were strongly associated with survival in BRCA patients (3). Peng et al. constructed a molecular prognostic score based on 23 genes that accurately predicted the overall survival of BRCA patients (4). In addition, various prognostic models were constructed between cancer types, and these features were shown to be more accurate in predicting clinical prognosis than assessed by traditional pathological and imaging methods (5–7). In the field of BRCA research, new prognostic models are not yet fully developed. Therefore, we included breast cancer-related genes in the construction of prognostic models to estimate novel strategies for predicting outcomes in BRCA patients.

Over time, immunotherapy has made more significant progress in other cancer types, including melanoma, kidney cancer, and lung cancer (8). In the early stage, due to the weak immunogenicity of BRCA, it is regarded as a “cold tumor”, and scholars believe that immunotherapy is difficult to make a big breakthrough in it. Still, in recent years, immunotherapy has made good progress in triple-negative BRCA, especially in metastatic BRCA (9–12). Therefore, finding an effective way to predict long-term survival and response to immune checkpoint inhibitor treatment in BRCA patients is critical (13).

In this study, weighted gene co-expression network analysis was used to screen out the genes associated with BRCA prognosis. The prognostic features were derived from univariate Cox regression and LASSO regression analyses performed on the TCGA BRCA training cohort. After rigorous validation in multiple cohorts, we demonstrated that the POLQ-related signature can effectively predict BRCA prognosis. By using the median risk score, we stratified the BRCA samples into high-risk and low-risk groups, which displayed distinct overall survival, progression-free survival, and disease-free interval, as well as differences in clinical characteristics, immune infiltration, response to ICI treatment, and chemotherapy drug sensitivity. To facilitate clinical application, we developed a nomogram that can guide BRCA treatment. These findings shed light on the immunological and prognostic significance of POLQ in BRCA and highlight the potential of its related biomarkers as promising targets for the diagnosis and treatment of BRCA.

## 2 Materials and methods

### 2.1 Data collection

Utilizing multiple datasets, including GSE16228, GSE20685, GSE20711, GSE42658, and GSE88770 from the Gene Expression Omnibus (GEO) database, you collected a total of 745 BRCA tumor tissue samples and 43 normal tissue samples as training sets. Additionally, 1089 BRCA tumor samples from TCGA were used as the testing cohort. To ensure accurate analysis, patients lacking important clinical information such as OS and relapse status were excluded, and data normalization was performed to mitigate batch effects.

### 2.2 WGCNA

By constructing a weighted gene coexpression network using the WGCNA R software package, you aimed to identify coexpression gene modules, investigate the relationship between the gene network and phenotype, and identify core genes within the network. Here’s an overview of the process we followed: Calculation of Median Absolute Deviation (MAD): You calculated the MAD for each gene in the BRCA gene expression profile. Selection of genes: The top 50% of genes with the smallest MAD values were excluded. This step helped filter out less informative genes. Removal of outliers: The “goodSamplesGenes” method from the WGCNA package was used to remove outlier genes and samples from the dataset. Construction of a scale-free co-expression network: Using WGCNA, you built a scale-free co-expression network. The Pearson correlation matrix and average linkage method were employed for pairs of genes. Weighted adjacency matrix construction: A power function (A_mn=|C_mn|^β) was applied to construct the weighted adjacency matrix. The soft threshold parameter, β was used to accentuate strong gene correlations and compensate for weaker ones. Topological overlap matrix (TOM): The weighted adjacency matrix was transformed into a TOM to estimate network connectivity. The hierarchical clustering method helped create a cluster tree structure for the TOM matrix. Module analysis: Dissimilarity of characteristic genes within the modules was computed. Tangent lines of the module tree were selected, and some modules were combined for further analysis.

### 2.3 Identification of DEGs and functional enrichment analysis

We identified 129 differentially expressed genes between the BRCA group and the normal control group using the “Limma” package. The selection criteria included a |logFC| ≥ 1 and p < 0.01. Next, you employed the “clusterProfiler” package for conducting the Gene Ontology (GO) function and Kyoto Encyclopedia of Genes and Genomes (KEGG) pathway enrichment analysis based on these DEGs. GO function analysis categorizes genes into different functional groups, while KEGG pathway analysis explores molecular interactions and networks within cells. These analyses provide valuable insights into the biological mechanisms underlying BRCA and can reveal potential therapeutic targets and interventions.

### 2.4 Random survival forest variable screening

Random Survival Forest (RSF) is an ensemble method that consists of a collection of randomly growing survival trees. The tree-building rules in RSF are similar to those of random forest, which is an extended methodology used for analyzing survival data. To identify prognostic genes in the training set, univariate Cox proportional regression models were initially employed. Then, 1000 classification trees were constructed using bootstrap samples. During the tree-building process, candidate variables were randomly selected at each node, and nodes were classified based on survival criteria such as survival time and truncation information. To determine the variables entering the model, gene screening was performed using exponential sequencing or gene occurrence frequency. Each decision tree within the random survival forest is a binary survival tree, generated following the top-down recursive splitting principle. This approach involves sequentially dividing the training set from the root node, which helps prevent model overfitting. Using RSF, researchers can obtain valuable insights into the prognosis of specific diseases, including potential prognostic genes and their impact on survival outcomes.

### 2.5 GeneMANIA analysis

GeneMANIA (http://www.genemania.org) is an excellent resource for constructing protein-protein interaction (PPI) networks and analyzing gene function and interactions. This user-friendly database enables researchers to visualize functional networks between genes and gain insights into gene behavior. The GeneMANIA website provides the flexibility to customize the data sources of gene nodes, including physical interaction, gene coexpression, gene colocalization, gene enrichment analysis, and predictions from other sources. By incorporating these diverse data sources, researchers can obtain a comprehensive understanding of gene relationships and their functional implications. In this study, we utilized GeneMANIA to construct a core gene network specific to ovarian cancer patients. This network serves as a valuable tool to investigate the potential mechanisms underlying the action of identified genes within the context of ovarian cancer. By visualizing and analyzing these gene interactions, we can gain insights into the functional associations and pathways involved in the disease. Make sure to carefully interpret the findings from the gene network analysis and consider additional validation strategies to strengthen the conclusions.

### 2.6 Analysis of immune cell infiltration

To assess various aspects of the tumor microenvironment (TME) in BRCA patients, we employed several algorithms. The ESTIMATE algorithm allowed us to evaluate the Stromalscore, Immunescore, and TMEscore. These scores provide insights into the levels of stromal and immune cell infiltration within the tumor. For a detailed analysis of immune cell types in the TME, we utilized the CIBERSORT algorithm. CIBERSORT is a widely used method that employs support vector regression to deconvolute the expression matrix of immune cell subtypes. This enabled us to quantify the levels of immune cell infiltration in each patient. To further assess the TME, we employed the MCPcounter algorithm. This algorithm generates abundance scores for eight immune cell types and two stromal cell types (including T cells, CD8+ T cells, cytotoxic lymphocytes, NK cells, B lymphocytes, monocytes, bone marrow dendritic cells, neutrophils, endothelial cells, and fibroblasts) based on the gene expression matrix. Notably, MCPcounter has been verified to exhibit a high correlation between estimated scores and actual cell scores when conducting quantitative validation. By evaluating the association between the risk scores (derived from the previously constructed prognostic model), gene expression levels, and immune cell infiltration, we aim to understand the interplay between the genetic signature and the immune microenvironment in BRCA. Additionally, we examine subgroup differences in immune checkpoint expression and immune function, providing valuable insights into potential immunotherapeutic targets and treatment strategies.

### 2.7 Development of the prognostic model

From a pool of 100 intersection genes, we conducted univariate Cox regression (uniCox) analysis to identify 18 genes that showed associations with prognosis. Subsequently, we employed the “glmnet” package for regression analysis, specifically utilizing LASSO-cox analysis, to construct a prognostic model. The risk scores for individual patients were calculated by summing the gene expression values multiplied by their respective gene coefficients in the model. This risk score served as an indicator of the likelihood of an adverse prognosis. To further analyze and visualize the patient data, we divided the patients into high and low-risk groups based on the median risk score. Utilizing the “stats” package (version 3.6.0), we performed Principal Component Analysis (PCA). This analysis aids in dimensionality reduction by transforming and clustering the patients’ gene expression profiles. Specifically, we initially applied a z-score transformation to standardize the gene expression data and then utilized the prompt function to obtain a reduced matrix representing the principal components. It’s worth mentioning that the selection of genes and the use of LASSO-cox analysis contribute to the construction of a robust prognostic model. Moreover, conducting PCA analysis allows for a comprehensive visualization of the patient data, which may reveal patterns or clusters related to prognosis.

### 2.8 Clinical significance analysis of the risk model

After excluding patients with missing data, we integrated their clinical information and risk score to conduct uniCox and multivariate Cox regression (multiCox) analyses. These analytical approaches allow us to assess the relationship between the risk score model and patient prognosis. To evaluate the accuracy of the risk score model as a prognostic predictor, we performed ROC analysis using the pROC package (version 1.17.0.1). The Area Under Curve (AUC) values were calculated to provide a quantitative measure of the predictive power. We also used the CI function of the package to determine the Confidence Intervals (CI) around the AUC values, providing a measure of the uncertainty associated with the predictions. By analyzing the AUC values and their corresponding CI, we can determine the final results and assess whether the risk score model can serve as an independent and reliable prognostic predictor. It’s important to note that further studies may explore alternative methods or consider additional variables to enhance the predictive capabilities of the risk score model. The pROC package is a valuable resource in this process, facilitating the calculation of AUC values and their associated confidence intervals.

### 2.9 Establishment of a predictive nomogram

In addition to the patient’s risk score and clinicopathological features, we employed the “rms” package to construct a nomogram. This nomogram combined multiple variables to visualize their relative contributions in predicting patient outcomes. To assess the prognostic predictive power of these clinical features, particularly for 1-year, 3-year, and 5-year OS, we performed ROC analysis. The ROC analysis allows us to evaluate the accuracy of the predictive models by examining the true positive rate against the false positive rate. To validate the predictive accuracy of the nomogram, we utilized calibration curves. These curves provide graphical representations of the agreement between predicted outcomes and observed outcomes. The incorporation of calibration curves allows for an assessment of the accuracy and reliability of the predictive model. It’s important to note that future studies may further explore alternative methods or consider additional factors to enhance the predictive capabilities of the nomogram. The “rms” package serves as a valuable tool in this process, facilitating the creation of comprehensive and informative prediction models.

### 2.10 Drug sensitivity analysis

The Genomics of Drug Sensitivity in Cancer (GDSC) database, accessible at https://www.cancerrxgene.org/, serves as a valuable resource for cancer drug sensitivity genomics. In our study, we utilized the R software package called “pRRophetic” to predict the sensitivity of tumor samples to chemotherapy. By applying a filter condition of p < 0.001, we determined the chemotherapy sensitivity for each tumor sample. The prediction process involved regression analysis to estimate the IC50 values for specific chemotherapeutic agents. To evaluate the accuracy of regression and prediction, we conducted 10-fold cross-validation tests using the GDSC training set. For all parameters, including the removal of batch effects using “combat” and averaging repeated gene expression, we opted for the default values. These steps enable us to mitigate potential biases and enhance the reliability of the predictions. It is worth noting that future research may explore alternative parameter settings or incorporate additional validation techniques to further refine the predictions. The GDSC database and the pRRophetic package offer a powerful combination for investigating and understanding chemotherapy sensitivity in cancer samples.

### 2.11 Cell transfection

Two distinct small interfering RNAs (siRNAs) targeting POLQ were synthesized by Ribobio (Guangzhou, China). The transfection protocols were executed following the manufacturer’s guidelines, using Lipofectamine 3000 (Invitrogen, USA). **Supplementary Table 1** contains the siRNA sequences for POLQ.

### 2.12 RT-qPCR

Total RNA was extracted from tissues or cell lines using TRIzol reagent (15596018, Thermo) and standard protocols were followed. Subsequently, cDNAs were synthesized using the PrimeScript™RT kit (R232-01, Vazyme). The Roche LightCycler 480 platform (Roche, GER) was employed to quantify gene expression levels, utilizing SYBR qPCR Master Mix (Q111-02, Vazyme). **Supplementary Table 1** contains the primer sequences, which were sourced from Tsingke Biotech (Beijing, China).

### 2.13 Cell counting kit-8 assay

Each well of a 96-well plate was seeded with 2000 treated cells. Following this, the cells were subjected to treatment with the CCK-8 labeling reagent (A311-01, Vazyme) and evaluated at various time points, including days 1, 2, 3, 4, and 5.

### 2.14 Wound healing

After achieving 95% confluency, the transfected cells were subsequently plated into 6-well plates. To produce a straight line, a sterile pipette tip with a volume of 200 μL was utilized, followed by gentle rinsing with PBS to remove any unattached cells and debris. Subsequently, the serum-free cell medium was replaced to maintain cell culture. Images were captured at 0 and 48 hours in the same location.

### 2.15 Transwell

A density of 2×10^4^ cells per well in 200 μL of serum-free medium was used to seed the cells in the upper chamber of a transwell plate. The upper chamber was either coated or uncoated with matrix glue (BD Biosciences, USA). The lower compartment was loaded with 700 μL of complete medium supplemented with 10% serum. After 36 hours of incubation, the cells were fixed, stained, and counted by microscopy. Images were captured for analysis.

### 2.16 Statistical analysis

All statistical analyses were performed using R language (Version 3.6). All statistical tests were bilateral, and *P <*0.05 was considered statistically significant.

## 3 Results

### 3.1 Identification of DEG sets associated with BRCA patients compared to normal women

**Figure 1:**
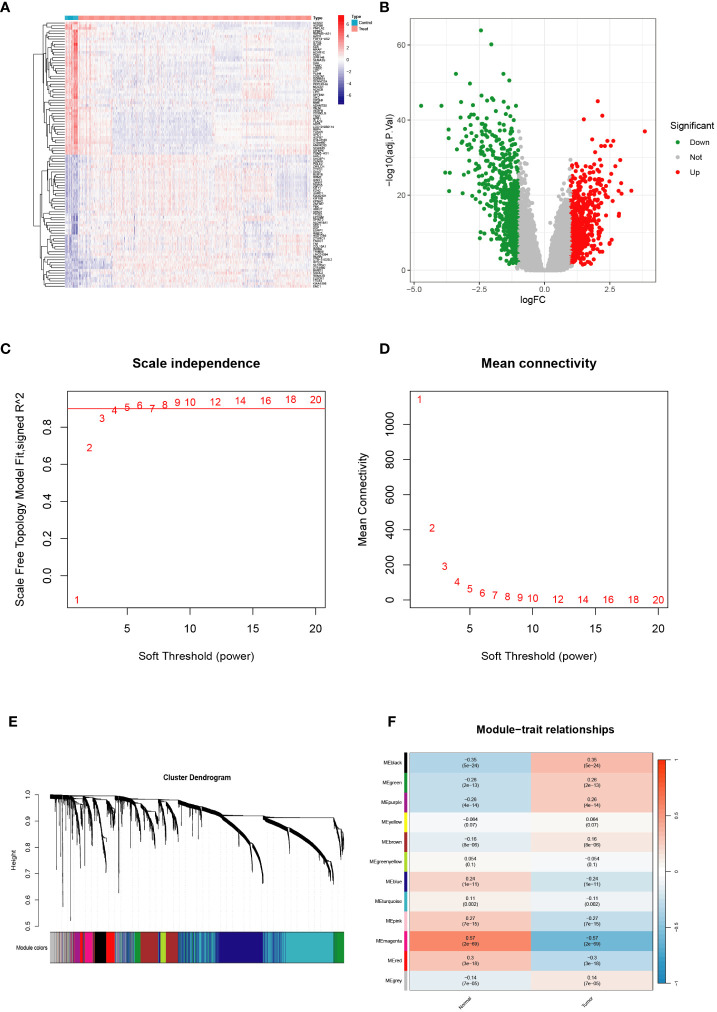
Validation of the hub module via weighted gene coexpression network analysis. **(A, B)** Differentially expressed genes are shown on the heatmap and the volcano plot for the five Gene Expression Omnibus (GEO) datasets. **(C, D)** The scale-free fit index and the average connectivity of soft threshold power and hierarchical clustering tree of genes based on topological overlap are confirmed for the five GEO datasets. **(E, F)** A total of 12 modules were obtained and the correlation of these modules between the normal group and tumor group for the five GEO datasets.

To establish our study cohort, we obtained gene expression profiles from five GEO datasets, comprising 745 tumor tissue samples and 43 normal control tissue samples, which served as the training cohort. Additionally, we utilized the TCGA-BRCA cohort, consisting of 1089 BRCA tumor tissue samples, as the validation cohort. In the training set, we identified genes that demonstrated significant differential expression (|logFC|>1 & p<0.01) (**Figures 1A, B**). We employed WGCNA analysis to construct a gene coexpression network for BRCA using the training set. Subsequently, we employed dynamic hybrid cutting to generate a hierarchical clustering tree, which facilitated the identification of gene modules. The tree branches represented groups of genes with similar expression patterns, with each gene represented as a leaf in the tree (**Figures 1C–E**). Furthermore, we successfully constructed twelve modules within the training cohort, and we identified the magenta modules as potential hub modules (**Figure 1F**).

### 3.2 Acquisition of intersection genes and molecular characteristics analysis in BRCA

**Figure 2:**
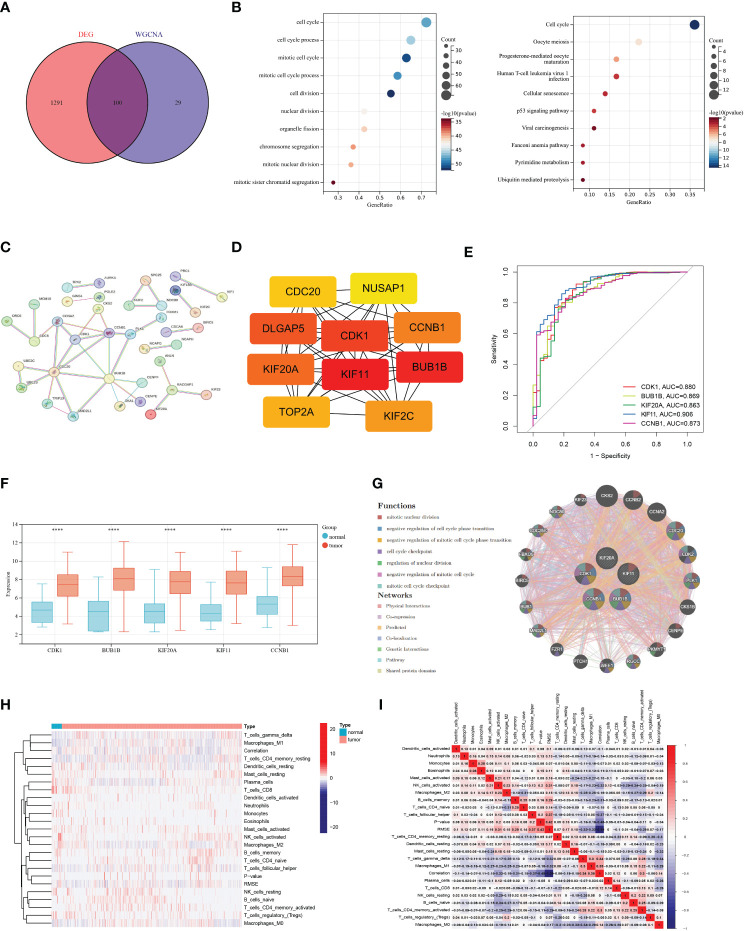
Acquisition of intersection genes and molecular characteristics analysis. **(A)** Venn diagram based on differentially expressed genes and hub gene set. **(B)** Gene Ontology and Kyoto Encyclopedia of Genes and Genomes analysis of intersection genes. (C) The protein-protein interaction network was acquired from the STRING database among intersection genes. **(D)** Hub genes in the intersection gene set. **(E)** Receiver operating characteristic curve of five hub genes. **(F)** Differential expression of hub genes in breast cancer (BRCA) and normal tissues. **(G)** The networks among five hub genes are based on the GeneMANIA database. **(H–I)** Immune cell infiltration analysis in BRCA and normal tissues. (P<0.0001 ****).

To verify the reliability of the genes obtained above, we performed a Venn analysis based on the DEG set and hub gene set data (**Figure 2A**). A total of 100 intersection genes were screened for GO and KEGG enrichment. GO analysis showed that these intersection genes are enriched in a variety of biological processes, including cell cycle, mitotic cell cycle process, cell division, nuclear division, and chromosome segregation. KEGG analysis revealed that cell cycle, oocyte meiosis, progesterone-mediated oocyte maturation, and p53 signaling pathway were enriched by these intersection genes (**Figure 2B**). In addition, we constructed PPI networks using an online tool (https://cn.string-db.org) to explore the association between the intersection genes. The results showed that CDK1, BUB1B. KIF11, KIF20A, and CCNB1 were hub genes (confidence score = 0.900) (**Figures 2C, D**). These hub genes are highly expressed in tumor tissues compared to normal control tissues and in the ROC curve, these hub genes displayed a pretty AUC value (**Figures 2E, F**), implying their potentially critical roles in BRCA. Furthermore, the Genemania network also indicated that the five genes have a close interaction in multiple biological functions, including mitotic nuclear division, negative regulation of cell cycle phase transition, and cell cycle checkpoint (**Figure 2G**). In immune cell infiltration analysis, we found that a variety of immune cells, including CD8^+^T cells, plasma cells, activated NK cells, and macrophages M2 were more abundant in normal tissues, while macrophages M1, T cells CD4^+^ memory resting, and γδ T cells were more infiltrated in tumor tissues (**Figures 2H, I**).

### 3.3 Development and validation of the prognostic model

**Figure 3:**
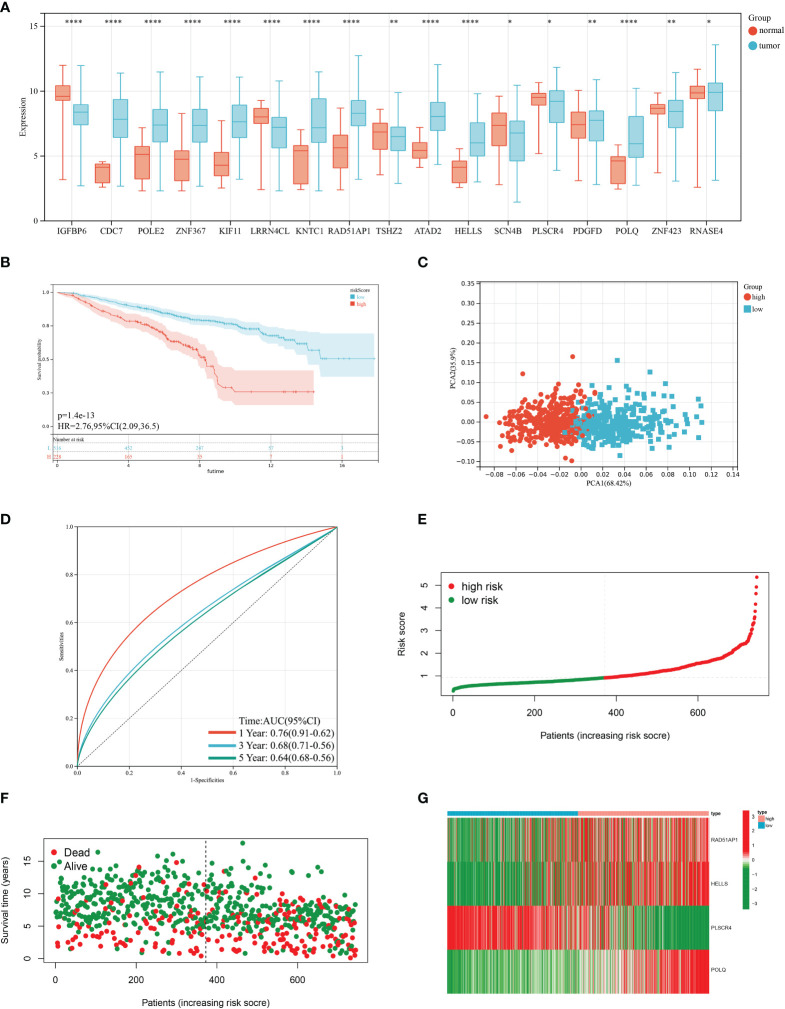
Construction of the prognostic model in the training cohort. **(A)** Differences in the expression of 17 prognostic-related genes among BRCA and normal tissues. **(B)** Kaplan–Meier analysis of the Overall Survival (OS) between high- and low-risk groups. **(C)** Principal Component Analysis based on the prognostic model. **(D)** Receiver operating characteristic curve to predict the sensitivity and specificity of 1-, 3-, and 5-year survival according to the risk score. **(E, F)** Ranked dot and scatter plots showing the risk score distribution and patient survival status. **(G)** Expression patterns of 4 selected prognostic genes in high- and low-risk groups. (*P*<0.05 *; *P*<0.01 **; *P*<0.001 ***, *P*<0.0001 ****).

**Figure 4:**
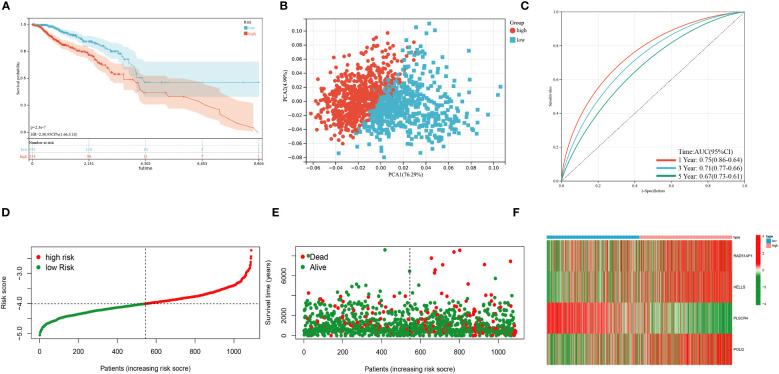
Validation of the prognostic model in the test cohort. **(A)** Kaplan–Meier analysis of the Overall Survival (OS) between high- and low-risk groups. **(B)** Principal Component Analysis based on the prognostic model. **(C)** Receiver operating characteristic curve to predict the sensitivity and specificity of 1-,3-, and 5-year survival according to the risk score. **(D–E)** Ranked dot and scatter plots showing the risk score distribution and patient survival status. **(F)** Expression patterns of 4 selected prognostic genes in high- and low-risk groups. (P<0.05*; P<0.01**; P<0.001***, P<0.0001****).

Based on 100 intersection genes, the researchers conducted a uniCox analysis and identified 18 prognostic-related genes, which were found to be highly expressed in tumor tissues (**Figure 3A**). They then developed a risk model to assess the prognostic predictive ability of these intersection genes specifically in BRCA patients. To establish the optimal predictive model, LASSO and multiCox analysis were performed on 18 prognosis-related differentially expressed genes. Ultimately, four genes (RAD51AP1, HELLS, PLSCR4, and POLQ) were identified, and the formula for the risk score was derived as follows: risk score = (0.171787939 * expression of RAD51AP1) + (0.180159626 * expression of HELLS) + (-0.35136865 * expression of PLSCR4) + (0.35452173 * expression of POLQ). Following the risk score calculation, the patients were divided into high-risk and low-risk groups based on the median risk score. Kaplan-Meier analysis demonstrated that low-risk patients had a better OS compared to the high-risk patients (**Figure 3B**). Additionally, PCA analysis showed a distinct separation of patients into high-risk and low-risk groups (**Figure 3C**). The prognostic model exhibited promising predictive ability with AUC values of 0.76 (95% CI = 0.91-0.62), 0.68 (95% CI = 0.71-0.56), and 0.64 (95% CI = 0.68-0.56) for predicting patients’ OS at 1, 3, and 5 years, respectively (**Figure 3D**). Moreover, there was an inverse correlation between the risk score and patient survival, as evidenced by the decreasing OS and increasing mortality rate with higher risk scores (**Figures 3E, F**). The expression heatmap in **Figure 3G** illustrates the gene expression patterns involved in constructing the prognostic model. Importantly, the prognostic model was validated in an independent cohort and showed good predictive power (**Figures 4A–F**).

### 3.4 Clinical correlation analysis of the prognostic model and construction of a nomogram

**Figure 5:**
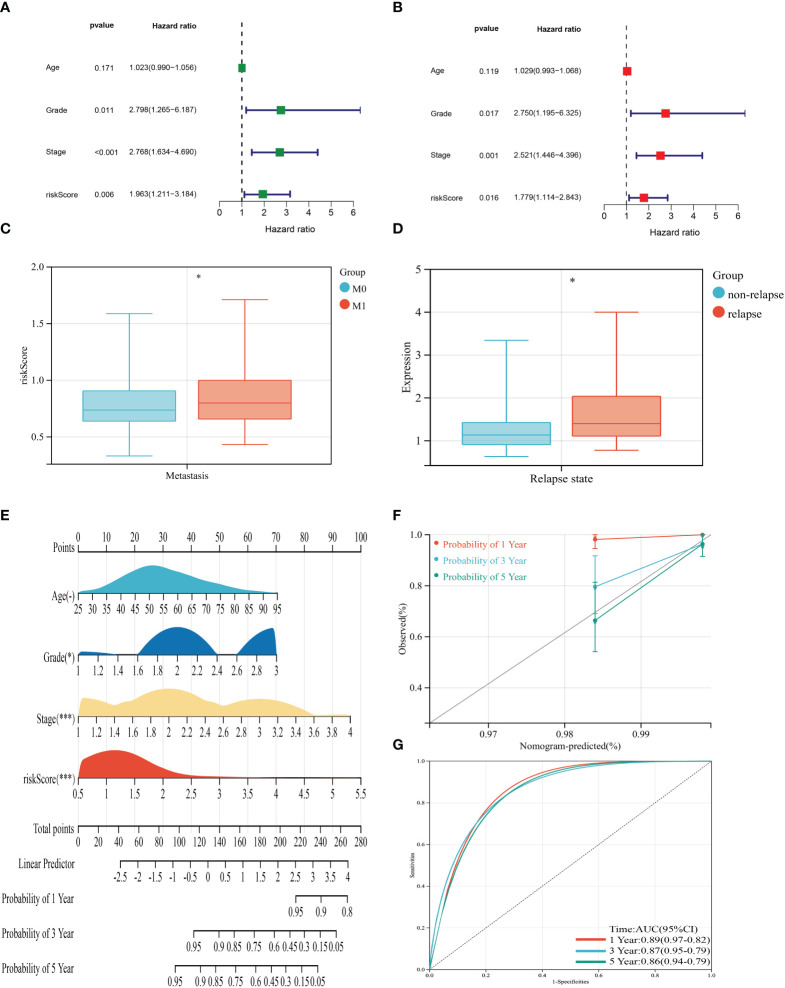
Clinical correlation analysis of the risk score and establishment of the prognostic nomogram. **(A, B)** uniCox and multiCox analysis showed the prognostic value of the risk score. **(C)** Correlation between risk score and tumor metastasis of BRCA. **(D)** Correlation between risk score and tumor relapse of BRCA. **(E)** Nomogram for predicting the 1-, 3-, and 5-year OS of BRCA patients in the entire cohort. **(F)** Calibration curve of the prognostic nomogram. **(G)** Receiver operating characteristic curves of the prognostic nomogram for 1-, 3-, and 5-year OS in BRCA. (*P*<0.05 *; *P*<0.01 **; *P*<0.001 ***).

UniCox and multiCox analyses were conducted to assess the independent prognostic value of the risk score (**Figures 5A, B**). The forest plot indicated that the risk score is comparable to tumor grade and tumor stage as an independent risk factor for predicting the prognosis of BRCA patients. Patients who experienced distant metastasis or tumor relapse exhibited higher risk scores (**Figures 5C, D**), suggesting a correlation between the risk score and increased risk of metastasis and poorer prognosis. To enhance predictability, a nomogram was developed based on clinical characteristics to estimate the 1-year, 3-year, and 5-year OS of BRCA patients (**Figure 5E**). The calibration curves in **Figure 5F** demonstrated the high accuracy of the nomogram in predicting the 3-year and 5-year OS of BRCA patients. Additionally, the receiver operating characteristic (ROC) curve in **Figure 5G** revealed the AUC values of the nomogram for predicting the 1-year, 3-year, and 5-year OS: 0.89 (95% CI = 0.97-0.92), 0.87 (95% CI = 0.95-0.79), and 0.86 (95% CI = 0.94-0.79), respectively.

### 3.5 Assessment of TME, checkpoints, and immune function in distinct groups

**Figure 6:**
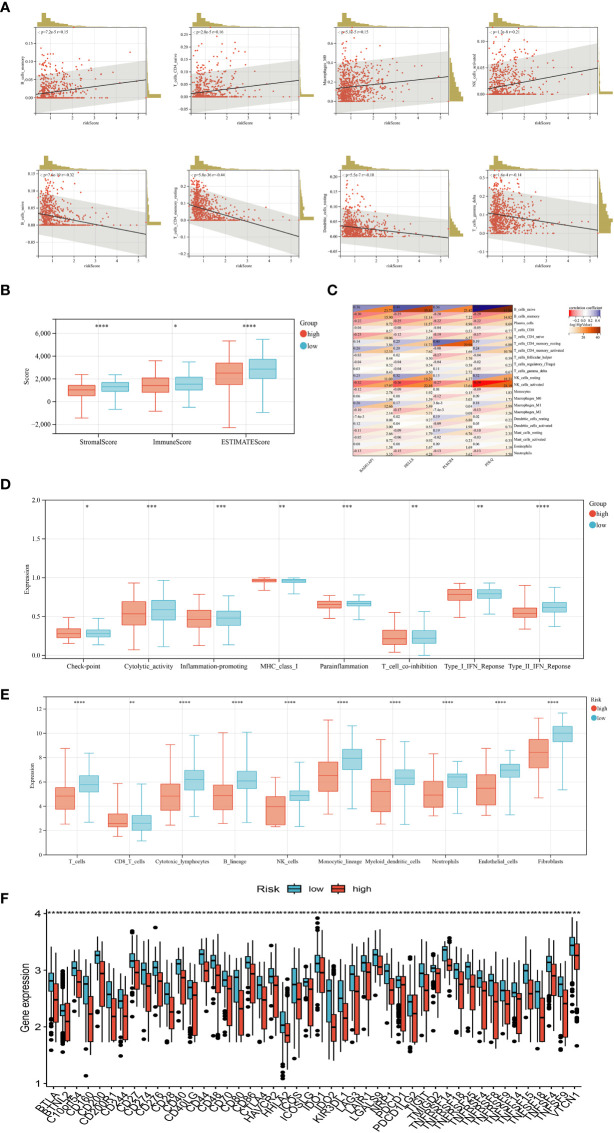
Evaluation of tumor microenvironment, checkpoints, and immune functions between the two groups. **(A)** Correlations between risk score and immune cell infiltration levels. **(B)** Correlations between risk score and tumor microenvironment scores. **(C)** Correlations between the abundance of immune cells and genes involved in the development of the prognostic model. **(D)** Assessment of differences in immune function between the two groups. **(E)** Abundance of 8 infiltrating immune cell types and 2 stromal cell types in the two groups. **(F)** Expression of 35 common immune checkpoints in the two groups. (*P*<0.05 *; *P*<0.01 **; *P*<0.001 ***, *P*<0.0001 ****).

In our study, we explored the correlation between immune cell abundance and the risk score. Our findings, as depicted in **Figure 6A**, revealed interesting associations between the risk score and different immune cell types. Specifically, the risk score positively correlated with memory B cells, naïve CD4^+^ T cells, macrophages M0, and activated NK cells. Conversely, it exhibited a negative correlation with naïve B cells, resting CD4^+^ memory T cells, resting dendritic cells, and γδ T cells. Furthermore, our analysis demonstrated that the risk score was linked to higher StromalScore, ImmuneScore, and ESTIMATEScore, as illustrated in **Figure 6B**. This suggests that the risk score may indicate increased stromal and immune activity within the TME. To delve deeper, we assessed the association between the genes utilized in constructing the risk score model and the infiltration levels of various immune cells. Notably, we observed significant correlations between the expression levels of certain genes, such as POLQ, and the abundance of specific immune cell types. For instance, the expression level of POLQ displayed a significant positive correlation with naïve B cell infiltration and a negative correlation with activated NK cell infiltration, as shown in **Figure 6C**. **Figure 6D** revealed significant differences in various immune functions between the high-risk and low-risk groups, such as T cell co-inhibition, immune checkpoint expression, cytolytic activity, and type I IFN response. The MCPcounter analysis in **Figure 6E** demonstrated that the high-risk group had decreased infiltration levels of endothelial cells, fibroblasts, and immune cells compared to the low-risk group. Moreover, by analyzing 35 common immune checkpoints including PD-1, PD-L1, CTLA-4, and LAG3 between the high and low-risk groups (**Figure 6F**), it was observed that the low-risk group exhibited lower levels of immune checkpoint expression.

### 3.6 Drug sensitivity analysis

**Figure 7:**
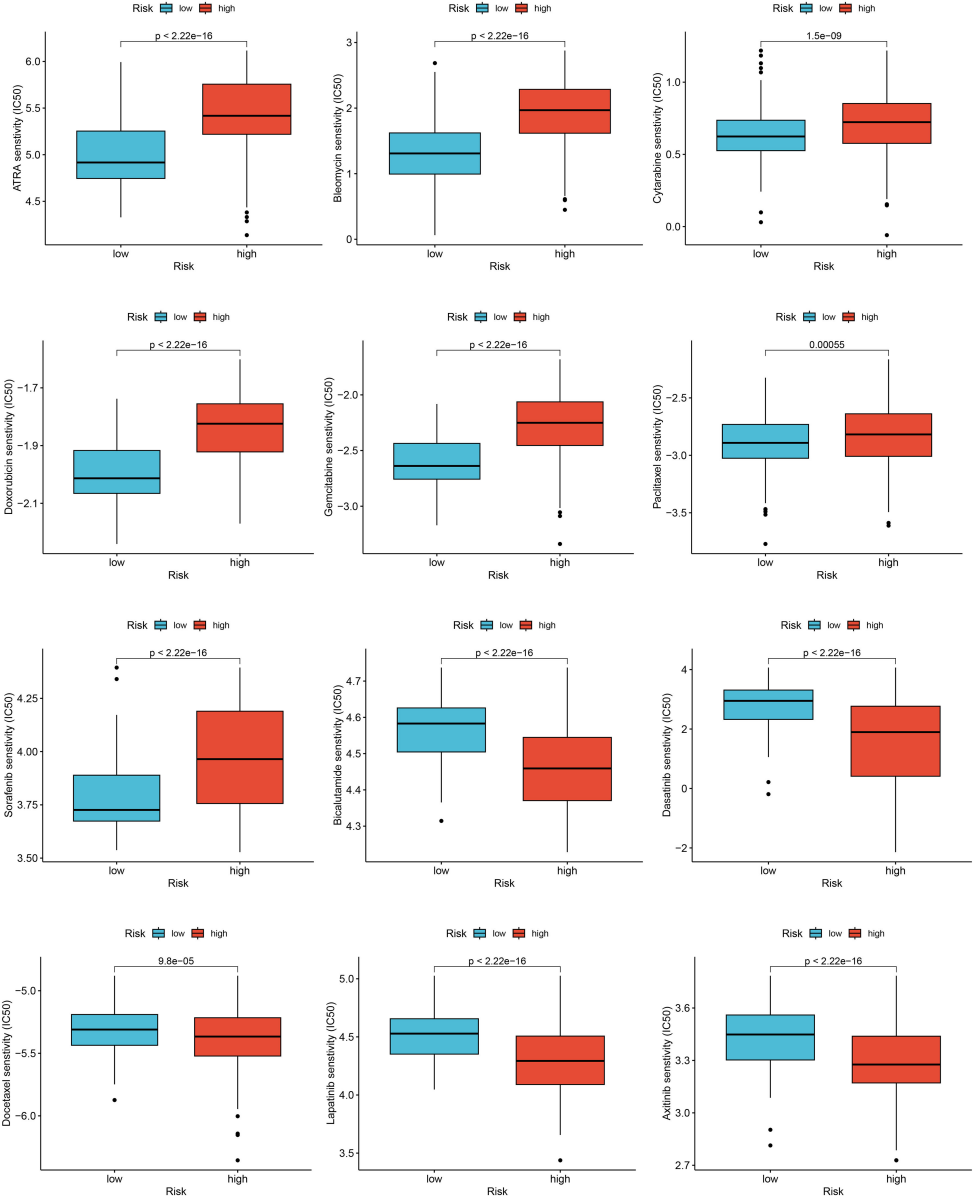
Relationships between risk score and drug sensitivity.

For evaluating the predictive power of the risk score in assessing clinical drug therapy sensitivity among BRCA patients, we employed the “pRRophetic” package to calculate the IC50 values associated with 138 drugs for each patient. Our analysis yielded intriguing findings regarding potential drug responses based on the risk score. Patients with low-risk scores exhibited promising indications of positive responses to several drugs, including All-trans-retinoic acid (ATRA), bleomycin, cytarabine, doxorubicin, gemcitabine, paclitaxel, and sorafenib. On the other hand, patients with high-risk scores displayed a greater likelihood of positive responses to bicalutamide, docetaxel, as well as various targeted therapy drugs such as dasatinib, lapatinib, axitinib, and other similar medications (**Figure 7**). Taken together, these outcomes suggest a noteworthy correlation between the risk score and drug sensitivity. Nonetheless, it remains essential to exercise caution and further validate these observations through rigorous validation studies. May these findings contribute to the advancement of personalized medicine approaches for better management of BRCA patients!

### 3.7 Core genes related to genetic alterations, TMB, and targeted therapy/chemotherapy in BRCA

**Figure 8:**
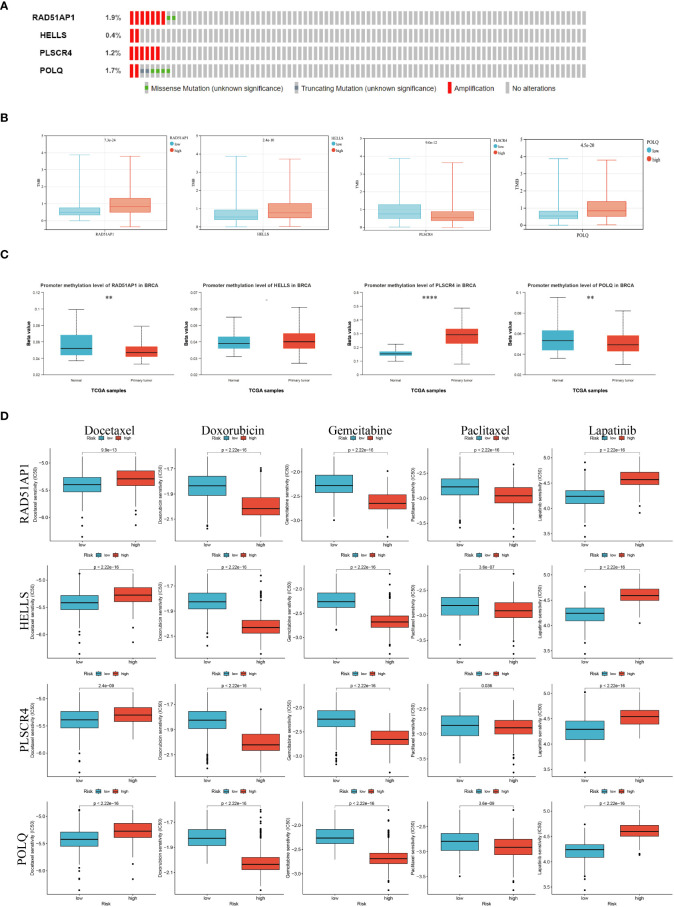
Epigenetic modification and genetic alteration of 4 key genes. **(A)**The DNA alteration of 4 key genes. **(B)** Tumor mutation burden of 4 key genes. **(C)** The DNA promoter methylation levels of 4 key genes are based on the Cancer Genome Atlas database. **(D)** The estimated IC50 values of docetaxel, doxorubicin, gemcitabine, paclitaxel, and lapatinib for 4 key genes. (*P*<0.05 *; *P*<0.01 **; *P*<0.001 ***, *P*<0.0001 ****).

To determine whether four genes exist in the somatic mutation frequencies in BRCA, we extracted the oncoprint profiles based on the cBioPortal database (http://www.cbioportal.org/). The somatic mutation frequencies of RAD51AP1, HELLS, PLSCR4, and POLQ were 1.9%, 0.4%, 1.4%, and 1.7%, respectively (**Figure 8A**). In addition, tumor mutation burden (TMB) is a very important and identifiable clinical biomarker for immunotherapy. We calculated the TMB of each sample, and after analysis, we found that there was a significant correlation between the expression levels of RAD51AP1, HELLS, PLSCR4, POLQ, and the TMB data. Specifically, high expression of PLSCR4 was associated with lower TMB. In contrast, high expression of RAD51AP1, HELLS, and POLQ was associated with higher TMB in BRCA (**Figure 8B**).

In addition, previous studies have shown that abnormal epigenetic modifications can drive tumorigenesis and resistance to treatment. We extracted DNA methylation profiles of these core genes in BRCA based on the TCGA database and the results showed increased methylation levels in BRCA compared to normal breast tissue (**Figure 8C**).

Taking into account the chemotherapy used in daily work, we evaluated the response of gene expression subtypes (low or high expression levels) to five chemotherapy agents and one ErbB-2 inhibitor: Docetaxel, Doxorubicin, gemcitabine paclitaxel, and Apatinib. Interestingly, low RAD51AP1, HELLS, PLSCR4, and POLQ may be more sensitive to docetaxel, apatinib, while high RAD51AP1, HELLS, PLSCR4, and POLQ may be more sensitive to doxorubicin, gecitabine, and paclitaxel (**Figure 8D**).

### 3.8 Biological function and POLQ expression in BRCA are confirmed

**Figure 9:**
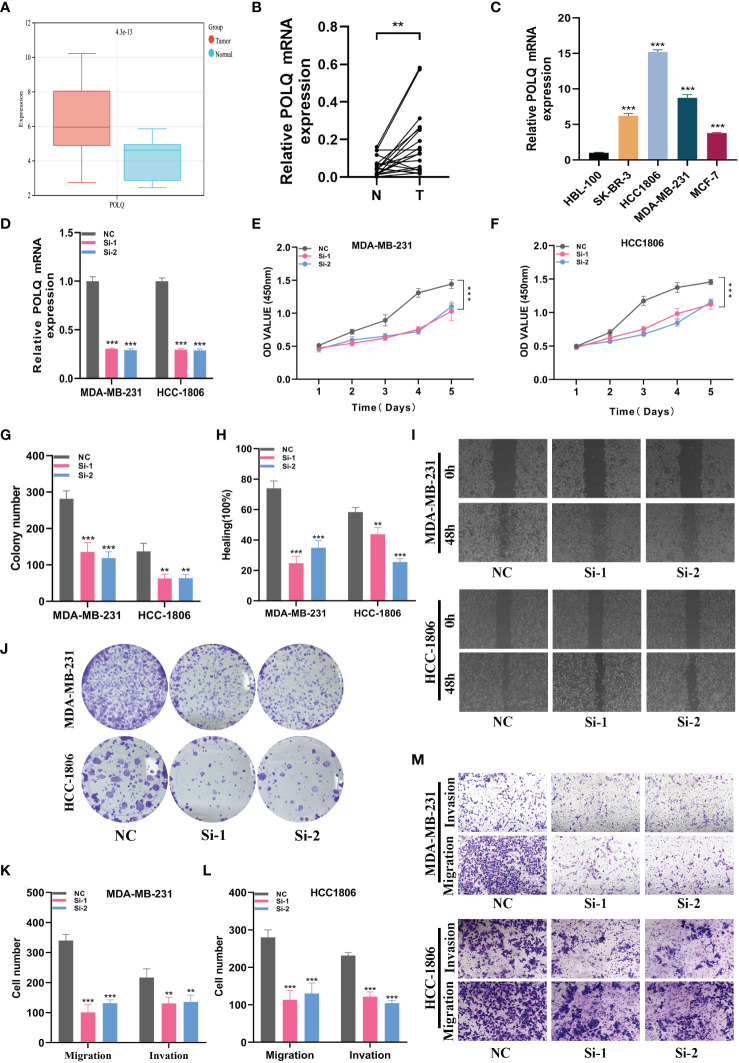
*In vitro* experiment about POLQ. **(A)** POLQ is highly expressed in breast cancer. **(B)** The expression level of POLQ is higher in breast cancer tissues. **(C)** The expression level of POLQ was higher in the MDA-MB-231 or HCC1806 cells. **(D)** Transfection efficiency of POLQ. **(E, F)** CCK-8. After POLQ knockdown, the proliferative ability of MDA-MB-231 or HCC1806 cell lines decreased significantly. **(G, J)** Clone formation. After POLQ knockdown, the proliferative ability of the two cell lines decreased significantly. **(H, I)** Healing test. After POLQ knockdown, the migration ability of MDA-MB-231 or HCC1806 cell lines decreased significantly. **(K–M)** Transwell assay. After POLQ knockdown, the migration and invasion abilities of MDA-MB-231 or HCC1806 cell lines were significantly decreased. (P<0.05*; P<0.01**; P<0.001***, P<0.0001****)

By doing *in vitro* tests, we furthered our understanding of POLQ ‘s role. Firstly, the results of bioinformatics analysis showed that the expression of POLQ in BRCA tissues was higher than that in normal tissues (**Figure 9A**). We verified the results in 20 pairs of clinical tissue samples and obtained consistent results (**Figure 9B**). To select suitable BRCA cell lines for POLQ knockdown assay, we verified the expression level of POLQ in 5 cell lines, and the results showed that the expression level of HCC1806 and MDA-MB-231 cell lines was relatively high (**Figure 9C**). Therefore, we selected these two cell lines for POLQ knockdown experiments and verified their transfection efficiency (**Figure 9D**). In CCK-8 studies, we saw that cells with POLQ knockdown displayed significantly decreased proliferative activity (**Figures 9E, F**). Colony-forming experiments also showed that the proliferation ability of melanoma cells was significantly reduced after POLQ knockdown (**Figures 9G, J**). Wound healing experiments showed that the migration ability of BRCA cells was significantly reduced after POLQ gene knockdown (**Figures 9H, I**). After the knockdown of POLQ, two cell lines significantly reduced their ability to heal, migration, and invasion (**Figures 9K–M**).

## 4 Discussion

Heterogeneity is one of the important characteristics of BRCA (14). Although most studies believe that BRCA is monoclonal in origin, due to multiple divisions, proliferation, and continuous evolution in the process of occurrence and development, epigenetic, genomic, and microenvironment changes lead to different phenotypes and biological characteristics of cells, resulting in heterogeneity (15). It showed different histological types, differentiation degree, cell proliferation rate, invasion and metastasis ability, and therapeutic responsiveness. Therefore, the accurate implementation of personalized medicine for BRCA requires further research and exploration (16, 17).

In our study, we used the WGCNA method to screen out BRCA-related genes that are specifically expressed in BRCA tissues. Subsequently, four genes were identified by difference analysis, univariate Cox regression, lasso regression, and multifactor Cox regression, and were further used to create a new risk profile. Patients were divided into high-risk and low-risk groups based on a median risk score. The difference in survival rate between the high-risk group and low-risk group was statistically significant, and the prognosis of the low-risk group was significantly better than that of the high-risk group (P<0.01). Validation analysis of TCGA and GEO databases showed that our prognostic model based on related genes could well distinguish BRCA patients. The forest map showed that risk score was an independent risk factor for predicting prognosis in patients with BRCA compared to tumor grade and tumor stage. We also found that BRCA patients who developed distant metastases or tumor recurrence had higher risk scores. Overall, based on our findings, a higher risk score implies a higher risk of metastasis and a worse prognosis. Compared to other existing BRCA prognostic models, such as apoptosis-related gene prognostic models (AUC at 1, 3, 5 years = 0.637, 0.701, 0.695), platelet-related prognostic models (AUC at 1, 3, 5 years = 0.639, 0.563, 0.596), and anoikis and immune-related gene prognostic models (AUC at 1, 3, 5 years = 0.521, 0.643, 0.695), our model shows more accurate predictive performance (18–20).

Immunotherapy is a new type of anti-tumor therapy that is completely different from the previous anti-tumor therapy (21). Under normal circumstances, immune cells are the protectors of our body kingdom, and the body’s immune system has an immune surveillance function, which can recognize, kill, and timely eliminate abnormal cells in the body (22). As abnormal cells, tumor cells can be recognized and eliminated by the body’s immune system under normal circumstances. Immunotherapy has also been carried out in a series of studies in BRCA, although its effect is not as significant as in lung cancer, melanoma, and other tumors, it has achieved a certain effect in triple-negative BRCA (23, 24), while the results in HR-positive/HER2-negative and HER2-positive BRCA are still immature or the efficacy needs further exploration (25). At present, the immune checkpoint inhibitor used in BRCA clinics is programmed death protein-1 (PD-1) programmed death protein ligand-1 (PD-L1), and other immune checkpoint inhibitors are still being studied and explored (26). The effective rate of immune checkpoint inhibitors is low, and most of them are combined with chemotherapy drugs. The research on the combination with other drugs is still underway (27). What we know is that one of the important reasons for the low efficiency of immune checkpoint inhibitors and their use in combination with chemotherapeutic agents in clinical treatment is that the immunotherapy-sensitive population cannot be accurately screened by the available means, whereas the TMB may drive effective anti-tumor immune responses and ultimately lead to a sustained clinical response to immunotherapy. Our results showed that the expression of genes involved in the model, such as POLQ, was significantly correlated with the TMB data in the BRCA patients, and that this group of patients with high POLQ expression levels was more likely to show better therapeutic effects to immunotherapy, which may provide a certain reference value for the individualized precision treatment of BRCA patients in the clinical practice. However, this needs to be confirmed by more real-world studies. Chimeric antigen receptor T (CAR-T) cell therapy is a personalized immunotherapy approach that has made some progress in BRCA treatment (28). Car-T cell therapy works by introducing a CAR that targets a BRCA-specific antigen into a redesigned T cell in the patient’s body, thereby activating and boosting the patient’s immune system to attack tumor cells. Vaccine therapy is a method of using specific antigens to stimulate the patient’s immune system to produce an anti-tumor immune response. In BRCA immunotherapy, researchers are developing various vaccines, including cancer vaccines, tumor polypeptide vaccines, and genetic vaccines, to activate the patient’s immune system to fight BRCA (29). In addition to the strategies mentioned above, there are several other BRCA immunotherapies under investigation. Examples being explored include the combination of immune checkpoint inhibitors, tumor-associated antigen (TAA) specific T cell therapy, and the use of immune promoters (30). While there have been some encouraging advances in BRCA immunotherapy, the effectiveness and safety of these strategies are still being evaluated in research and clinical trials. Therefore, for specific patients, it is still necessary to discuss and make decisions in detail according to individual circumstances.

In our study, we further calculated the correlation between immune cell abundance and risk score. We found that risk scores were positively correlated with memory B cells, primary CD4^+^ T cells, macrophage M0, and activated NK cells, and negatively correlated with primary B cells, resting CD4^+^ memory T cells, resting dendritic cells, and gamma-delta T cells. Given the important role of initial CD4^+^ T cells, macrophage M0, and other immune cells in immunotherapy, our prognostic model has a certain guiding significance for the immunotherapy response of BRCA patients. Our results also found that the genes involved in the model construction were significantly correlated with the infiltration levels of most immune cells. For example, POLQ gene expression levels were significantly positively correlated with naive B cell infiltration and negatively correlated with activated NK cell infiltration. At present, the immune checkpoint inhibitor used in BRCA clinics is programmed death protein-1 (PD-1) programmed death protein ligand-1 (PD-L1), and other immune checkpoint inhibitors are still being studied and explored. We analyzed 35 common immune checkpoints such as PD-1, PD-L1, CTLA-4, and LAG3 between the high and low-risk groups, and found that the expression level of immune checkpoints in the low-risk group was lower. This may indicate a better response to immunotherapy in the high-risk group. However, the efficacy of immune checkpoint inhibitors is low in single-drug treatment, and most of them are combined with chemotherapy drugs, and the study of combination with other drugs is still ongoing. Therefore, clinicians must identify individualized treatment at an early stage, as sensitive drugs vary from person to person. To find chemotherapy drugs that are more sensitive to high-risk populations, we perform drug sensitivity analysis to develop specific drugs for high-risk populations.

DNA repair is indeed crucial for maintaining genomic stability and preventing the development of cancer. The evolving understanding of the DNA damage response pathway has expanded the possibilities for therapeutic approaches in oncology. It is becoming increasingly clear that genomic instability in cells caused by defective DNA damage responses contributes to the development of cancer (31, 32). On the other hand, these defects can also serve as a therapeutic opportunity (33, 34). Targeting various components of the DNA Damage Repair (DDR) pathway, such as PARP, ATM, ATR, CHK1, WEE1, and DNA-PK, has led to the development of DDR-targeted drugs, some of which are currently under clinical study (35, 36). Currently, inhibitors of these DDR components, some of which are under clinical study (37, 38). It’s also interesting to note the potential synergy between DDR inhibitors and conventional cancer therapies, as well as their correlation with immune checkpoint inhibitor response, which promotes the exploration of combination therapies. These advancements in DNA repair-targeting drugs are increasingly playing a significant role in the field of tumor therapy (39, 40). Drugs that target DNA repair pathways are showing an increasing role in the field of tumor therapy (33, 39, 41).

POLQ is a large protein composed of helicase (HD) and polymerase domains (PD), and deletion of either leads to synthetic lethality of HR deficiency (42). POLQ is a promising target in cancer therapy, and POLQ inhibitors are being actively developed by the scientific and industrial communities (43). On August 5, 2021, Artios Pharma published the clinical registration of the First-in-class drug ART4215 on the clinical trials website. ART4215 is the world’s first highly selective oral small-molecule targeted POLQ inhibitor in the Polθ polymerase domain to enter clinical studies. On August 10, 2022, Artios Pharma announced that it has initiated a Phase II study of ART4215 in combination with the PARP inhibitor talazoparib in BRCA-deficient BRCA. POLQ helicases and POLQ polymerase inhibitors have been developed, and these POLQ inhibitors (POLQi) have specific effects on killing BRCA-deficient cells (44), but the specific mechanism of action of POLQ is unknown. More clinical trials are needed to confirm the promise of POLQ inhibitors in BRCA patients, especially those at high risk.

The study looked for monitoring and predictors of immunotherapy in BRCA patients, such as immune-related markers or gene expression characteristics. This will help determine which patients are suitable for immunotherapy and provide individualized treatment decisions. By delving deeper into immunotherapy for BRCA, we can reveal the interaction between the immune system and the tumor, develop more accurate and effective treatments, and improve patient outcomes and survival. However, the prognostic model we constructed through bioinformatics requires further external validation on independent datasets to verify its predictive performance and generalization ability. In addition, the application of the prognostic model in clinical practice should fully consider the actual clinical environment, feasibility, and interpretability. At the same time, BRCA is a dynamically changing disease, and biomarkers and clinical characteristics of patients may change over time. Prognostic models need to be able to account for this variation and provide real-time, valid recommendations in treatment decisions.

## 5 Conclusions

In this study, transcriptomics and proteomics were combined to conduct a comprehensive analysis. These data are integrated to conduct in-depth research, break through the limitations of a single omics study, conduct a joint analysis of different omics data, dig for more meaningful information in the limited data, build the body regulatory network, and deeply understand the regulation and causality between various molecules. The immunological and prognostic significance of POLQ in breast cancer was explored systematically. Notably, we advocate effective prognostic signatures based on POLQ-related genes. Our findings provide a novel and accurate classification and treatment strategy for breast cancer patients.

## Data availability statement

The datasets presented in this study can be found in online repositories. The names of the repository/repositories and accession number(s) can be found in the article/**Supplementary Material**.

## Ethics statement

The studies involving humans were approved by Ethics Committee of Nanjing Medical University. The studies were conducted in accordance with the local legislation and institutional requirements. The participants provided their written informed consent to participate in this study.

## Author contributions

WC: Conceptualization, Writing – original draft, Writing – review & editing. YK: Data curation, Writing – original draft, Writing – review & editing. WS: Formal analysis, Writing – original draft, Writing – review & editing. QH: Methodology, Writing – original draft, Writing – review & editing. JC: Software, Writing – original draft, Writing – review & editing. SP: Data curation, Methodology, Writing – original draft, Writing – review & editing. YM: Methodology, Software, Writing – original draft, Writing – review & editing.
