# Supplementary material for: A new 4-gene-based prognostic model accurately predicts breast cancer prognosis and immunotherapy response by integrating WGCNA and bioinformatics analysis
Source: Front Immunol. 2024 Feb 2;15:1331841. doi: 10.3389/fimmu.2024.1331841 (PMC10869553; doi:10.3389/fimmu.2024.1331841)
Supplement: Supplementary file 1 [file Table_1.docx]

| **Oligonucleotides** | **Nucleotide sequence (5'-3')** |
| --- | --- |
| **siRNA** |  |
| Scramble control | GCUUCGCGCCGUAGUCUUA |
| Si-POLQ-1 | CGGGCCTCTTTAGATATAAAT |
| Si-POLQ-2 | CCTTCAATCTTGCTTGCGAAA |
|  |  |
| **Primer** |  |
| GAPDH | GGCCTCCAAGGAGTAAGACC (forward) |
|  | AGGGGAGATTCAGTGTGGTG (reverse) |
| POLQ | CATCTTGGTTCGGCCACTCT (forward) |
|  | CTTTCACACAACGGGCCAAG (reverse) |
|  |  |

**Table S1. Oligonucleotides used in research**
